# Physical Activity during the Retirement Transition of Men and Women: A Qualitative Longitudinal Study

**DOI:** 10.1155/2021/2720885

**Published:** 2021-08-30

**Authors:** Marco Socci, Sara Santini, Sarah Dury, Jolanta Perek-Białas, Barbara D'Amen, Andrea Principi

**Affiliations:** ^1^Centre for Socio-Economic Research on Ageing, IRCCS INRCA-National Institute of Health and Science on Ageing, Via Santa Margherita 5, 60124 Ancona, Italy; ^2^Faculty of Psychology and Educational Sciences, Department of Adult Educational Sciences, Vrije Universiteit Brussel (VUB), Pleinlaan 2, 1050 Brussels, Belgium; ^3^Institute of Sociology, Jagiellonian University, Grodzka 52, 31-007 Crakow, Poland

## Abstract

The retirement transition is a major life change affecting people's lifestyles and behaviors, including those in relation to physical activity (PA), which is a key component of active ageing. Previous research analyzing the effect of retirement on PA levels has shown mixed results, and few studies investigated this issue in a gender perspective, thus, highlighting a need of knowledge in this respect. Aims of this study focused on the experience of PA during the retirement transition were to understand typologies of PA and possible changes in these typologies, to identify behavioural types relative to PA practice and levels, and to distinguish the main drivers and barriers for practicing PA associated with the different behavioural types. A further goal of the study was to investigate the abovementioned aims considering differences between women and men. Analyses were carried out within a three-year qualitative longitudinal study (2014-2016), which explored the individual experience of PA during the transition from work to retirement of 24 women and 16 men in Italy, with interviews carried out one year before and one and two years after retirement. Results show that preferred PA for both women and men was walking, along the transition to retirement. Over time, several participants replaced physically demanding activities with lighter ones. Six behavioural types were identified, describing individuals who incremented, started, or maintained the same level of PA, people who decreased PA levels or stopped it, and individuals who had a fluctuant behavior towards PA, or who had never practiced it. In general, poor health represented the main barrier to PA. For men, the main driver to PA was its effects on body shape, while for women, socialization/networking. In order to stimulate a more effective promotion of PA during the retirement transition, policy implications were discussed in light of the results obtained.

## 1. Introduction

### 1.1. Physical Activity as a Component of the Active Ageing Paradigm

According to the World Health Organization (WHO), active ageing is “the process of optimizing opportunities for health, participation and security to enhance quality of life as people age” ([1], 12), while healthy ageing is defined as “the process of developing and maintaining the functional ability (i.e. people's capabilities of being and doing what they have reason to value) that enables well-being in older age” ([2], 28) The active ageing paradigm [3, 4], which is focused on health benefits and well-being as outcomes of activity [5], contributes to healthy ageing of older people through participation in a wide range of activities, among which physical activity (PA). By practicing PA, older individuals can facilitate the maintenance and improvement of their physical capacity, enabling them to live healthier and more fulfilled years [3, 6].

There is clear evidence that practicing regular PA contributes to the primary and secondary prevention of several chronic diseases, as cardiovascular diseases, hypertension, depression [7, 8], diabetes, cancer [9, 10], osteoporosis [11], preventing also the risk of falls [12], and delaying the course of both dementia and Alzheimer's disease [13]. Being engaged in PA is also associated with a reduced risk of decrease in muscle strength, loss of muscle mass [14, 15], and premature death [16, 17]. Similarly, there would be a relation between the amount of PA and health status, such that the most physically active people would be the least at risk, by practicing a healthy lifestyle [16, 17].

Despite PA being crucial for a healthy and fulfilling life, there is a lack of insights and consensus on how life events such as the retirement transition affect older adults' PA, as well as on what barriers and drivers there are for practicing PA after the retirement transition (e.g., [18]). In order to fill some of the knowledge gaps in this area, the present longitudinal study is aimed at exploring the management of PA during the transition from work to retirement of older people in Italy, focusing on differences between men and women, as well as on drivers and barriers that might stimulate or hinder PA. Shedding light on these aspects can help designing appropriate policies and measures to promote PA during the retirement transition.

The context in which PA takes place and displays are necessary to have a full understanding of PA by unfolding the interplay with broader social, structural, and cultural environment, also known as the ecological variables. At the microlevel, PA in older age has been found to reduce risks to develop chronic diseases, as well as physical and cognitive functional decline [19]. It has also been found to increase life satisfaction, quality of life, and the satisfaction with the ageing process of individuals (e.g., [20, 21]). PA is also important for better physical and mental health, well-being, development of personal resources, increase of social contacts, and maintenance of independent living [2, 16, 22].

At the meso level, PA has been suggested to have a positive influence on the local community and society, by promoting social interaction and cohesion, since being engaged in PA increases opportunities for socialization and networking for people (e.g., by joining sport clubs, etc.), even in older age [17].

At the macrolevel, healthier and more engaged older people may contribute to save public spending, e.g., in health and social care [3, 23–25].

### 1.2. The Retirement Transition and Physical Activity: Mixed Evidence from the Literature

The retirement transition is considered a major life event affecting people's daily routines, lifestyle, and health behavior. Working in the labour market has a number of important functions for the individual, such as providing identity, structure, social stimulation, collective purpose, and activity [26]. This transition has been conceptualized as unfolding over three main phases, chronologically: the preparation for retirement while the individual is still working; the worker-retiree transition; and the adjustment to retirement [27]. The latter phase could be a period that can vary depending on individual circumstances [28] and relating to getting used to the changed aspects of life by achieving psychological comfort with the condition of the retired individual [29]. In light of this, studies dealing with the retirement transition must consider all three main stages, to shed light on the pathways to retirement. Many people may look forward to retirement while working, however, it may not be easy to adjust or adapt to the new role and circumstances, even if people engage in certain activities such as PA [30].

Two possible mechanisms, as adaptation (i.e., adjusting to the loss of work role) and exploration (i.e., retirement as opportunity to engage in activities in line with personal values), might play a role in explaining planning for being engaged in PA after retirement, as has been suggested in research focusing on these mechanisms as possible factors that can drive older workers to make plans for being engaged in paid work or volunteering after the retirement transition [31].

However, it is still not clear how retirement shapes and affects PA behaviors of older individuals. Previous research on PA levels during retirement has produced unclear and mixed results about the change. Not only about the change in time of PA levels but also about the positive or negative effects of retirement on older people through practiced PA (e.g., [20, 32–39]). Thus, there is no consensus on this issue [18].

When people retire, in line with the activity theory [40, 41], they often need to find other activities to replace work. First, due to the need of daily-time reallocation, in this perspective, retirement may mean opportunities for increasing PA, since the time previously spent at work can be used in this way [42]. Along these lines, Henning and colleagues [42] argued that PA (as well as other activities) can offer alternative roles outside the workplace to focus on, which should ease retirement adjustment. Other research pointed out that engaging in recreational/leisure-time PA (e.g., walking, yoga, and fishing) could be a way to occupy newly free time or a part of a new daily routine replacing the former working-day routine [37].

Accordingly, Lahti and colleagues [20] found that the retirement transition was associated with increased moderate-intensity PA (e.g., walking) and overall PA (i.e., moderate and vigorous PA) [20], while another study found a very little increase in leisure PA (i.e., sports, games, and other exercises) [35]. According to other research, retirement was associated with an increase of sport activities and exercise [32] or increased overall PA [34]. Systematic reviews of cross-sectional and longitudinal quantitative and qualitative studies, on the one hand, found that recreational/leisure-time PA increases after retirement; on the other hand, no clear pattern emerged for overall PA [36, 37].

The latter may mean that PA may also be managed in a perspective of continuity [43, 44] with no particular increase or decrease of the level of PA practiced. In this perspective, a high level of preretirement engagement in PA may have a positive impact on postretirement engagement in PA [42]. Lifelong attitudes of participation in PA can be a strong driver and a prerequisite for older people's PA after the transition to retirement. Yet, also in a continuity perspective, it has to be considered that lifelong patterns of physical in-activity often might persist even after retirement, thus, individuals who did not engage in PA during childhood and/or adulthood or later may not be engaged in PA after the retirement transition [45, 46]. Accordingly, Slingerland and colleagues [33] found no relationships between retirement and sport or leisure time PA (e.g., walking).

Additionally, and this may be read in a disengagement perspective [47], PA might decline as individuals are ageing [48, 49] and especially after the retirement transition, when the work-related PA gets lost, e.g., walking to the workplace [20]. In accordance with this perspective, some studies (e.g., [38, 39]) found that PA levels decreased, during the retirement transition. However, mixed evidence remains at that point.

### 1.3. Physical Activity during the Retirement Transition: Drivers and Barriers

It has also been observed that PA levels may swing during the retirement adjustment. Retirement is subdivided in different phases, each with its distinct PA patterns. This implies that later phases of retirement (i.e., after a considerable time after the retirement transition, once the individuals have settled in their retirement patterns) may involve decreased levels of PA [18]. Van Dyck and colleagues [50] argued that specific types of PA (e.g., cycling or other leisure-time activities and household PA) might increase during the transition to retirement and decrease (e.g., cycling) or remain the same (e.g., household PA) at a later stage [50]. The authors of this study concluded that for specific PA types, older adults tend to lapse into old habits, once they are accustomed to retirement.

The latter implies that the previous theories may be too simplified to explain the retirement transition and adjustment [5]. As far as PA is concerned, it may not be just a matter of continuity, of replacing activities, or of disengagement. The complexity represented by interactions between many factors, among which individual expectations, family circumstances, health, and environmental opportunities offered, among others, determines an increased diversity of retirement pathways and of interindividual differences concerning behaviors towards PA, during and after the retirement transition [5, 51, 52].

Accordingly, the resource-based dynamic perspective on the retirement adjustment postulates that change in retirement adjustment is a result of changes in social, emotional, financial, physical, cognitive, and motivational resources [53]. In this perspective, resources assume a major role in determining PA during and after the retirement transition, in the sense that the availability or not availability of certain resources, influences PA levels of individuals, and resources may be considered drivers or barriers to PA, depending on their availability.

Motivational resources seem to play a major role, in this respect, in which they depend on preferences and responsibilities of older individuals, and the way in which they prioritise PA compared to other aspirations or plans for their retirement [5, 18, 54]. Some studies (e.g., [36, 55, 56]) identified the expected benefits for health and well-being and broader social benefits like social networking and new relationships or opportunities for companionship [36], as the main key reasons for motivating people to engage in PA during the retirement transition. In some cases, even if individuals are aware of the positive health consequences of PA, they do not have a sufficient motivation to adopt PA behaviors. Accordingly, lack of motivation (in terms of laziness, lack of willpower, etc.), negatively influences PA, in the long term [45, 57, 58].

Another identified motivational reason to engage in PA was the perspective of new personal challenges, for instance, the wish of learning new skills, or to engage in competitive team sports [59] that might contribute to promote the individual perception of self-esteem and a sense of accomplishment, as well as to increase personal fitness and body shape [36, 59, 60]. Innovative opportunities for promoting PA behaviors among older adults are already available with use of healthy lifestyle technologies, such as wearable fitness devices and mobile health applications/devices [61]. For example, a research carried out in Switzerland [62] shows that 20.5% of people over 50 years of age use mobile devices for PA tracking, and that the interest for these new technologies is higher among active and younger older adults (50 to 64 years). However, it has been observed that the use of wearable and mobile health devices (e.g., activity trackers, smart watches) can motivate older adults to practice more PA in the short term, but the influence of such devices does not ensure the maintenance of the PA in the long-term [63, 64]. Nowadays, more promising than PA trackers seem to be the virtual coaching systems [65], especially if based on embodied conversational agents, e.g., avatar, that may ensure a more natural, human-like human-machine interaction [66], therefore, being more motivating than wearable devices.

As an important barrier to PA, some older people may simply not be interested in PA [58]. It could be the case of individuals that do not perceive the benefit of PA for personal health and well-being, or that do not like to do PA, preferring to invest their time, once retired, in other activities [36, 58].

A further important barrier influencing PA and PA levels is bad health status. Poor health conditions or health decline negatively influences PA during the retirement transition [51, 67].

Moreover, despite the supposed increased availability of time once retired, a crucial resource to engage in PA remains time availability. Some retired people can actually have more free time available, and they can use it (being a driver) even for engaging in PA. However, some other retirees can have little time available due to several reasons, as for example, family and caregiving commitments, voluntary work, and hobbies, thus, limiting the time to devote to PA [36, 60].

Other studies pointed to other factors that prevent people from practicing PA during and after the transition to retirement or that contribute to its reduction, e.g., the lack of lifestyle planning prior to retirement, living alone, limited social network, and (bad) weather/seasonal reasons [5, 68].

### 1.4. Physical Activity during the Retirement Transition: A Gendered Perspective

Few studies examined gender differences in PA and PA changes during the retirement transition, and their results were in some cases conflicting [37, 50]. To our knowledge, no study specifically dealt with gender differences in PA typologies, during the retirement transition.

Barnett and colleagues [37] discovered by means of a systematic review analysis that during the first stage of retirement, there is a slightly higher increase in leisure-time PA in men, than in women. Another study reported that moderate-to-vigorous PA (e.g., gardening) decreased in recently retired men, while it increased in recently retired women [50]. Other research [69] showed an increase in PA for both men and women after retirement, even though this increase is mainly experienced by people retiring at the official statutory retirement age and not by early retirees, irrespectively of gender [70]. Pedron and colleagues [71] found that women who retire early tend to improve significantly their PA levels, however, differently to what happens to men who retires early, probably not enough to compensate for the decrease in work-related activities (e.g., women may experience an increase in body mass index—BMI).

Despite the fact that women tend to retire earlier than men (e.g., in 2018, the OECD average retirement age was 63.5 years for women and 64.2 years for men, however, with quite huge differences among countries [72]), a study showed that retiring at older ages (e.g., 65 years and over) was associated with greater increases in PA during retirement transition compared to those retiring younger than 60 years of age, without specific differences among females and males, even though the latter tend to be more engaged in PA than women during the retirement transition [73].

Concerning motivational drivers for PA engagement in retirement, the need to socialize seems to play a role especially for women [59, 60]. The latter might miss social interactions of the working environment, and once retired, they may choose to join gyms and sport clubs for socializing, reducing loneliness, and establishing social networks. A motivational factor privileged by men seems to be the need to find new personal challenges through engagement in PA, thus, contributing to improve their fitness or body shape [59].

With respect the PA motivational driver of opportunity for companionship, one study found no gender differences [74], whereas Dawson and colleagues [75] reported this motivation as more important for female than for male respondents. Supporting this view, having fewer possibilities of companionship, was more important for women, than for men, as a barrier for PA engagement in later life and after retirement [76].

### 1.5. Aims of the Study

The results described above are not clear; and especially concerning what and in which way, specific drivers and barriers influence PA patterns during the retirement transition of men and women, and what is the impact of this in terms of PA experiences and pathways in time. This has been mainly due to the fact that previous studies in this field have been designed with a cross-sectional and/or retrospective approach, with implicit bias and limitations as a consequence of this [18, 77]. In some cases, where people retired several years beforehand, memories may have faded thereby risking distortions in recollections of feelings, and the past could be interpreted in light of subsequent experiences and life events. Most of previous studies also fail to capture the dynamic and the trajectories of PA after retirement transition, since PA behavior can change during retirement. Thus, it is important to explore the views of individuals who are approaching retirement and/or who have recently retired, e.g., up to two years after the retirement event, as this study did. Individuals at later stages of retirement, as those studied in most previous studies, may have already settled into a more habitual pattern of behavior and may also not be as receptive to programs aiming to change/stimulate their PA behavior.

The main aim of this study is to provide an understanding of the individual experience of PA of women and men, during the retirement transition. The present qualitative study has a longitudinal design, with individual interviews carried-out at three time points: the first one on the cusp of retirement, followed by another interview at one year after retirement, and the third interview two years after retirement. This study design allowed to study and to answer the following strategic research questions:

(RQ1) Are there differences between women and men, concerning types of PA practiced during the retirement transition?

(RQ2) Relative to PA levels, which types of individual behavior can be identified, during the transition from work to retirement? Are there differences between women and men?

(RQ3) Which are the main drivers and barriers for practicing PA associated with the different behavioural types identified during the retirement transition? Are there differences between women and men?

## 2. Materials and Methods

### 2.1. Recruitment Strategy and Inclusion Criteria

This study is based on the outcomes of the extending working life (EWL) longitudinal three waves research, carried out in Italy between 2015 and 2017. It involved a nonprobability purposive convenience sample [78, 79] of 40 individuals (24 females and 16 males) in the cusp of retirement, with the aim of shedding light on how retirement could impact on different individuals' life realms, i.e., health and well-being, social life, economic condition, assumption of alcohol, smoking, nutritional habits, and PA. This paper reports the findings concerning the PA practiced by older people during the retirement transition.

The main sample inclusion criteria were to be full-time employed and to be eligible to retire in 10-12 months. Participants were also purposively selected in order to ensure the diversity of the sample in terms of marital status (i.e., in couple, widowed, or single), income group, educational level, age, gender, and work typologies (sedentary, standing, physical, and heavy manual), so attempting to provide as broad a perspective as possible of the heterogeneity of the retirement experience, albeit the small sample of this qualitative study. Subjects were excluded if they were working part-time.

Participants were screened by a recruitment agency based on the abovementioned criteria, and then older employees matching the inclusion criteria were contacted by phone by three researchers skilled in qualitative interviews. Individuals who accepted to take part in the study were provided with and asked to sign a written informed consent to data treatment in accordance with the Italian legislation on privacy and data protection. In addition to the baseline, older people were contacted at two other points in time: one year and two years after the retirement date. There was no drop-out because all 40 individuals accepted to participate to the two follow-ups. Approval by the ethic committee was not mandatory nor advised by the Italian national legislation, because this was an observational (nonexperimental) study.

### 2.2. Sample Description

The mean age of the sample at the baseline was 60.4 years (min. 52/max. 66). The interviewed individuals were living in Northern and Central Italy (Emilia-Romagna and Marche regions). At baseline, all men (16) and more than half women (16) were in a loving relationship and lived in couple; two women were single, and one of them divorced, while five were widowed. Over the other two waves of the study, one man divorced, and one single woman got in couple.

### 2.3. Data Collection and Analysis

A planned prospective longitudinal qualitative interview design [80, 81] was carried out.

Data were collected through a semistructured topic-guide interview whose items at baseline were investigating the working situation, reasons for retirement, plans, and expected changes for the life after retirement concerning health and lifestyle (mainly nutrition, smoking, and alcohol assumption), social life (i.e., family and friendship networks, inclusion in and participation to social activities), and PA. For the latter, which is the main objective of this analysis, some open-ended questions were asked to respondents, such as, for example, “What do you do to be physically active?”; “How many times a week do you practice PA?”; “Are you satisfied with your PA levels?”. Subsequent interviews investigated the evolution of the situation during the retirement transition and adjustment, by exploring the same areas and asking the same questions over the second and third data collection times but opening every interview by asking if respondents wanted to highlight any event that occurred since the previous interview and that had had an impact on their life and retirement. Respondents reported, for example, one grandchild's born, the onset of an illness, one child's wedding, and partner's retirement. One hundred and twenty semistructured interviews (40 per three waves) were digitally recorded, transcribed verbatim, and analysed both deductively and inductively by two researchers separately, who discussed results with a third researcher and obtain consensus. This process was followed in order to minimise personal bias and interpretation errors, so assuring validity and reliability [82].

The study's trustworthiness, i.e., credibility, transferability, dependability, and confirmability [83], was ensured by tracking the internal analysis processes such as for example frequent debriefing sessions and constant reflective commentary among researchers about data coding, categorization, and interpretation [83–85].

Data were analysed with the method of qualitative content analysis [86], with the support of MaxQda software (version 2020) for the qualitative analysis. Specific topics and contents related to PA were filtered out of the textual material. Chunks of text have been named by categories and subcategories which were inductively developed and systematized in a code tree.

In order to build types having internal homogeneity and presenting aspects of heterogeneity towards other groups [87], we identified older individuals' levels of PA.

Once recognized that there is no consensus on a univocal method for defining or describing levels of activity based on self-report surveys [88], as it was the case in this study, the categorization adopted in this paper was inspired by the International Physical Activity Questionnaire (IPAQ). The latter quantitative tool measures three types of activities [89]: walking, moderate-intensity (activities that take moderate physical effort making breathe somewhat harder than normal), and vigorous intensity (activities that take hard physical effort making breathe much harder than normal, e.g., aerobics, fast bicycling, and running). Levels of PA are based on frequency (on a weekly basis) and duration (time spent in the activity). The three categories adopted by the IPAQ are high level, moderate (medium) level, and low level [90].

In light of the fact that the IPAQ the medium level corresponds broadly to at least one hour of vigorous intensity activity or at least 2.5 hours of moderate intensity activity or walking per week, and that it was not possible to reproduce exactly the same measurement through open questions during qualitative interviews; in this study, the medium level was categorized as at least about one hour and half of “vigorous” activities (e.g., gym, dance, swimming, and running), at least 2 hours and half “light” activities (e.g., cycling at a regular peace), or 5-9 kilometers of walking per week.

Less of this was considered as “low” level of activity, while more of this was considered as “high” level of activity.

When reporting findings below, quotations include the ID of respondents, their gender and age at baseline, as well as the wave when the interview was carried out.

## 3. Results

According to the IPAQ, Table 1 describes the level of PA of the sample at the baseline.

Most of the interviewed men declared low level of PA before retirement, while a considerable number of interviewed women (i.e., 10) did not practice PA while working.

**Figure 1:**
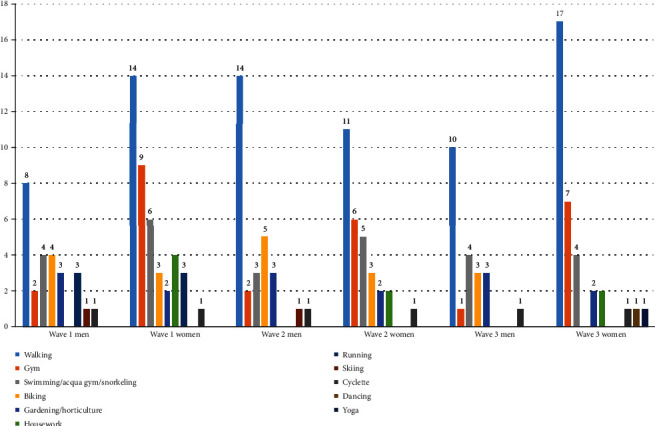
Typologies of PA done by older adults in transition to retirement, by gender (possible more than one answer per interviewee).

Answering RQ1 (i.e., “Are there differences between women and men, concerning types of PA practiced during the retirement transition?”), the PA typology preferred by older individuals of the studied sample, at baseline resulted to be walking, both for men and for women. The second most common PA among women was gym: one third of them do exercises at gymnasium or at home, while just two men out of 16 liked to do this type of activity. Among men, apart from walking, other common PA practices were biking (4) and swimming/snorkeling during summer (4) (Figure 1).

Given this starting point, every change over the three study waves, in typology of PA, and in its intensity was monitored through the data emerging from the participants' answers.

Over the course of the three waves, the respondents, both men and women, tended to replace more physically demanding activities with physically less demanding ones, for example, biking and running were replaced by walking, gym by dancing, and swimming by yoga. Regardless of the kind of PA left and the one started, for example, respondent number 6 can be considered emblematic of this shift to lighter PA along the retirement transition. At wave 1, she said “I run during the lunch-break twice a week” (ID6, female, 60, W1).

After retirement in wave 2, she referred to the following: “I gave up running and I started attending the gym: I go twice a week for two hours”.

And then in wave 3, she said

“Going to the gym was too strenuous. Now I walk mainly, at least one hour a day”.

So walking is the first choice of women and men in the sample. Indeed, the number of walkers increased over time. Retirees referred to like walking because this activity can be regulated on their needs (e.g., time availability, strength, and environment), and they can do it in company or alone depending on people preferences and so it can be fun and pleasant:

“I created a group of people who like walking like me. Every morning I meet these friends and we walk 2 hours and half in different places, from time to time” (ID22, male, 59, W2).

Gym remains an activity preferred especially by women even after retirement, because it can be flexible in times, as described by the following woman:

“I also go to the gym at least three times a week, without having set days, I go there in the middle of the day because there are fewer people, or in the evening” (ID30, female, 62, W3).

To answer to RQ2, investigating PA levels in relation to possible behavioural types and possible gender differences, the three-points in time analyses allowed to identify six behavioural categories: those who have the common characteristic that they always practiced PA in their life and they further increased it after retirement (i.e., increasers); those who had never done PA before retirement and who began practicing it after retirement (i.e., starters); those who did not modify their PA level when they retired: they practiced low PA when they were workers, and they continued at the same level when they were retirees (i.e., continuers). The three point in time analysis also evidenced cases of an up and down trend: individuals increased and decreased, or vice versa, their PA level during the retirement transition (i.e., fluctuaters). Other identified categories were “decreasers” (i.e., those who decreased PA levels year by year or completely gave up practicing PA) and people who never (i.e., neither before, not after retirement) practiced PA (i.e., inactive). The first three categories (i.e., increasers, starters, and continuers) would imply a positive impact of retirement on PA level. The remaining three categories (i.e., fluctuaters, decreasers, and inactive) would imply a less positive, if not negative, impact of it.

As shown in Table 2, for both women and men, the retirement transition may determine a considerable variety of paths, related to PA levels of older women and men. The results obtained by analyzing the sample under study seem to suggest that during retirement, PA levels of men (9 increasers and 3 continuers out of the whole 16 men investigated) may benefit more than PA levels of women (12 cases among increasers, starters, and continuers; 12 cases in the remaining three behavioural categories). As regard to the individual categories, the one of increasers is the most numerous for both women and men, while men are not represented among starters.

When behavioural types are related to the educational level of the studied individuals (Table 3), it is sound that most of the increasers have a low educational level and all the fluctuaters an intermediate one. There are not decreasers with a high educational level.

In order to answer RQ3, for each behavioural type as categorized above, drivers and barriers to PA have been investigated, by gender (Table 4).

### 3.1. Increasers

This group included nine men and six women of our sample. For these individuals, practicing PA represents a healthy habit that they have always had, the latter also being the main driver identified for this group of people, who during retirement increased PA since they had more time available for practicing it. The increasers (both men and women) did not mention any special obstacle to PA.

This is illustrated in the following comments of a man explaining that when he was working, he sometimes went out for a walk and rarely rode the bike. After retirement, he started going to the swimming pool as he liked to swim, and he saw the benefit of this activity (in terms of increased physical well-being). So, he started swimming twice a week:

“I am doing more PA than last year. I am trying to organize myself for well-being, to try to improve performance and to feel comfortable as I get older […] I like to go to the pool and so I signed up: I am going twice a week for an hour in the morning: I found benefits from this activity and I spend my time fruitfully” (ID25, male, age 61, W2).

Another example of increase in activities came from a woman who was already very active before retirement. In fact, both at wave 1 and 2, she declared to go to the gym once a week, to practice water aerobics once a week, to attend a dancing course another day per week, and to walk with friends during the weekend. Interestingly, over time, since she felt her energy decreased with age, she replaced water aerobics that become too physically demanding, with yoga. In the following quotation, she explained the reasons of such a high level of PA, by highlighting that PA has ever been a healthy habit for her:

“PA is very important! Not only for the enthusiasm I feel in doing it, as for the benefit it brings to the body. Apart from dancing, other kinds of PA are a burden for me. But still, I do it… for my health” (ID30, female, age 62, W3).

The following quotation is from a woman who continued walking whenever she could, despite she had pain to her knees, because this activity gave her a sense of freedom:

“When I can ... sometimes I walk with my grandchild in the stroller. I can't do more because my knees are gone. I walk almost 3 kilometers. [...] Walking makes me feel free” (ID29, female, age 59, W3).

Thus, practicing PA is a precise and aware choice, as highlighted by the following quotation from a man who walked or biked for long distances every day:

“I am more active than the last year because I want to be so” (ID15, male, age 65, W3).

Within this category, a special driver to PA that in our sample was present for women and was the willingness to meet people and to socialize as depicted by a woman who, after retirement, started walking every morning for at least one hour and went to the gym twice a week:

“I go to the gym and we are a group of women: we have some good chats” (ID34, female, age 62, W2),

Then, in light of the above, we can say that, although respondents from the increasers group experienced those that could be interpreted as barriers, such as for example the ageing process, the loss of physical energies or physical pain, they did not live these circumstances neither refer to them as barriers. Conversely, they adapted the typology of PA to their new physical condition and continued being active so demonstrating a good capability of adaptation along the ageing process. However, at baseline, when they were still working, many of them, both men and women, complained about the lack of time for doing more PA. Reasons for the mentioned lack of time in W1 were gendered: men could not increase PA mainly due to work commitments, while women mainly due to the difficulty in finding a balance between work and family duties, as depicted by the following quotations from a man and a woman, respectively:

“The main obstacle to PA when I was working, were work commitments and the work time. Now I am more active. I worked harder to increase physical activity and exercise for my health.” (ID05, male, age 61, W3).

“When I came back home from work in the evening, I had to do housework, and I took care of my daughter and of my father in law, so I had no time nor energy for doing more PA. Now I have more time and I can keep some of it for myself” (ID35, female, age 57, W3).

### 3.2. Starters

Whereas no men were found in this category; five women can be labeled as “starters.” The drivers of PA in this case are mixed between more available time, companionship, and willpower (and especially the latter), while the barriers during working life are represented by the counterparts of the drivers, i.e., lack of time, loneliness, and laziness. The women of this group did not report any barriers to PA after retirement, but conversely, they mentioned several barriers they experienced when they were still working. Since drivers and barriers are strongly interconnected in this group, in the cases reported below, we tried to highlight how the same elements that were barriers before retirement became drivers after retirement, i.e., how the lack of time became more time availability; the lack of companionship was replaced by companionship and the excessive laziness ended to be the impulse for starting practicing PA.

Building further on the above, a woman explained that due to the lack of work-life balance, she experienced lack of time and experienced this as the main obstacle when she was still working. Once retired, she could spend the freed-up time on PA. At wave 1, she said

“The main obstacle to practice PA relies in the work commitments that lead me to concentrate in the afternoon a whole series of activities that do not always leave me the time to go to the gym e.g. family care and housework” (ID33, female, age 60, W1).

One year after retirement she changed her behavior towards PA and said

“Now I have more free time, that I can spend in doing more physical exercise” (ID33, female, age 60, W2).

She confirmed the same concept at wave 3.

Another barrier to PA for the women of this group was the lack of company when they still were employed. This woman did not practice PA when she was working because she did not like to do it alone, and she did not find anyone to do it with her:

“I am basically lazy and I think that I would be more motivated if I can find someone who can come with me to the gym, but I did not find yet” (ID28, female, age 66, W1).

Once retired, she started going to the gym with her daughter and walking with her husband, so a driver has been to find the right companionship:

“The gym is the main activity. It was good that my daughter signed me up because otherwise I would have become much lazier” (ID28, female, age 66, W3).

The following represents an emblematic case of willpower and voluntary to healthy ageing. At wave 1, this woman referred that she had never practiced any type of PA due to her laziness:

“I would have time for physical activity. The problem is that I am very very lazy!” (ID32, female, age 59, W1).

Then, at wave 2, she had started walking twice a week saying:

“I understand that it's good for me. Aside from the fact that I have to do it for my health, I also want to do it (ID32, female, age 59, W2).

Finally, at wave 3, she had started attending a gym and she said

“I joined the gym for health reasons: I would have never imagined it! I was so lazy!” (ID32, female, age 59, W3).

### 3.3. Continuers

This group includes three men and one woman. Both for men and for the woman, and although they were able to continue practicing a low level of PA, the main obstacle to increase the level of it after retirement is represented by poor health. Poor health condition can in turn influence a situation of laziness; despite that, PA was considered as very important for health and aesthetic. The following comment reflects how poor health has affected their PA:

“Back pain limits me a lot. For about a couple of years I have had almost a handicap due to my back problems” […] the reasons why I always did physical activity? The first reason is keeping fit ... number two is the aesthetic aspect ... number three is that I feel good after having done physical activity” (ID14, male, age 52, W2).

These older adults with physical health problems evidence additional motivations to keep on doing the same level of PA. For instance, PA is a way to spend time with friends or with the partner:

“Sometimes I don't really want to do physical activity, but luckily a friend of mine with which I often bike is there, and he stimulates me, because if it were up to me, apart from stretching, it could stay a whole week without doing anything” (ID14, male, age 52, W3).

“Sometimes I want to walk but I don't feel like going alone, so I try to involve my husband, who doesn't want to. I should make up my mind and go walking alone” (ID16, female, age 63, W3).

People afferent to the continuer type demonstrate persistence, stubbornness, and resilience. In fact, although they did not increase PA, they maintained the same level of PA despite some potential barriers, e.g., health issues and lack of companionship.

### 3.4. Fluctuaters

The five individuals of this group (one man and four women) went through sudden events over time, thus, influencing PA levels, at least for a (more or less short) period of time. For the only man of this group, the main driver to PA was the awareness of the importance of PA for health. Conversely, the main barrier was poor health due to an accident that negatively influenced the interviewees' physical health, as underlined by the following quotation:

“I have a serious problem with my foot and this prevent me from practicing PA. I have to be careful, I can't exaggerate with walking. Nevertheless, I know that I must walk for blood pressure and so I try to walk two or three times a week for about 30 minutes” (ID19, male, age 61, W2).

Nevertheless, at wave 3, he said

“I am walking less than the last time we met, because I have pain to my foot [..] Every morning I went out and I tried to walk at least for 20 minutes but, as soon as I feel pain I have to stop walking” (ID19, male, age 61, W3).

Among the women of our sample, the main driver to PA was the fact that they liked it very much and they struggled not to totally give up, despite obstacles, so they just reduced and then increased again it (or vice versa).

Conversely, for women with a fluctuating behavior towards PA, the main obstacle to it was represented by caregiving responsibilities due to worsening health conditions of a family member:

“I planned to do a lot more sport activity, for example to go to the gym more often, to start other activities. However, my physical activity decreased, because my mom got sick with bronchopneumonia. After that, also my husband got sick. Then in March and April I had to look after to my granddaughter every day. In the end, now I got knee pain. In short, let's say that last year I was a lot more active” […]. I like running very much and if I do not go to the gym I go into crisis! Last week I could not exercise for three days and I could not even sleep because of it” (ID06, female, age 60, W3).

### 3.5. Decreasers

Two men and four women of the sample belong to this group. Among men, the main obstacle to PA seems to be laziness and lack of interest and motivation:

“I was able to be more determined before…until 2 years ago I run 3 times a week, even for one hour and half. Then I slowed down and gained weight, struggling to regain a better shape. In short, it didn't go well, I'm not happy. Last year I was already in a worsening phase and I haven't improved. I would like to do more in this area” (ID12, male, age 64, W2) […].

“I lost my rhythm a bit […] Maybe I was also a bit lazy, but now, in the next few days I would like to start again doing some physical activity” (ID12, male, age 64, W3).

Women belonging to this category mainly motivated their inactivity with poor health conditions:

“Till last year I used to take beautiful walks, even for one hour, after dinner. Now, I can't do them anymore because I'm afraid the knee won't hold. I am afraid it will get too inflamed” (ID31, female, age 57, W3).

Neither men nor women in this category identified any special driver of PA.

### 3.6. Inactive

Inactive individuals (one man and four women) have never practiced PA. The man at wave 1, defined himself as “lazy,” he said that he never has been interested in practicing PA and he confirmed this statement in the following interviews:

“I am lazy and I do not like gym basically. This is the only reason why I do not practice PA” (ID11, Male, age 64, W2).

Among the female study participants belonging to this group, the barriers to PA are a mix of poor health condition, lack of interest in PA, and caregiving duties, case by case to different extents. For example, the following quotation is from a woman suffering from asthma. She mentioned asthma as the first barrier to PA in the first and in the second interview. In the third interview, she said that the second obstacle to PA was the lack of company:

“I cannot do physical activity due to my asthma” (ID16 female, age 63, W1) […].

“Asthma is the main obstacle to my physical activity” (ID16, female, age 63, W2).

“I walk very rarely because I continue having problems with my asthma. To be honest, I could walk slowly, but I don't like doing it alone, then I try to involve my husband but he doesn't like walking and so he never come with me and at the end I give up!” (ID16, female, age 63, W3).

Another woman, caring for three older persons with long-term care needs (her mother with Alzheimer's disease, her father with multiple chronic diseases and a blind aunt cohabiting with her), said

“The three older persons I am caring for prevent me from any kind of physical activity. I would like to walk for two hours, for having respite from care, to free my mind, but I can't because of them. They limit my freedom” (ID38, female, age 62, W2).

The association between, retirement, health status and physical activity.

Regardless of the type of behavior towards PA, health status particularly influenced the study participants' capability of practicing PA, so this aspect deserves to be examined more in detail. Several interviewees with poor health, documented by specific diagnoses (e.g., herniated disc, ankle broken, asthma, and diabetes) or referred by respondents based on symptoms they were experiencing (e.g., pain to knees or overweight) at baseline or at wave 2, tended to gradually decrease PA levels because the lack of PA made them feeling weaker and weaker and unfit both physically and mentally, and this condition ended to demotivate them at a later stage. The case of interviewee number 4 can be taken as an emblematic example.

“Herniated disc ... in the back, I always have had this problem. I no longer have big problems with it, however I'm afraid my health could worsen again, in case I start to go to the gym. But now I would like to try again, because otherwise… movement, just gymnastics, I don't do it ...” (ID04, female, age 60, W1).

“The main barrier to physical activity is laziness, because if I wanted I could do not only water aerobics, but also swimming and walking more ... but for now let's say it's okay, let's do one thing at a time” (ID04, female, age 60, W2).

“I wanted to walk more, but… I became lazy….” (ID04, female, age 60, W3).

Again, about health, retirement had a positive impact on the general health and well-being of some study participants, and the better health condition became a driver for increasing PA levels. The case of interviewee number 2, belonging to the “incremental” type, seems to be a good example. He was 61 at the baseline and he carried out a sedentary work. As a long-lasting smoker, he suffered from chronic obstructive pulmonary disease and from the stagnation of lymph in the legs (i.e., lymphedema) that made them swell a lot. At wave 1, this interviewee weighed 149.5 kilos. One year after retirement, he said:

“I have stabilized my blood pressure. Blood sugar has dropped, cholesterol is below 200, heart is fine, weight is 18.5 kilos less, breathing has improved because I decreased the number of cigarettes ... the pains in my legs have dropped, ... in short, since last year everything has improved significantly. Compared to last year, it is much better. And all of this has positive effects on my mood too […] and my wife and I also managed to make love again” (ID02, male, age 61, W2).

Well-being and physical health continued improving more and more over time and at wave 3, the interviewee said

“Now my health conditions have improved significantly. Compared to two years ago, things have completely reversed ... even as a desire to live ... The pains I had two years ago are very distant memories, fortunately I stabilized my blood pressure, halved the dosage of medicines, I walk better, I lost almost 42 kg, I followed a “not heavy“ diet. Retirement was really a turning point I am very satisfied. In my opinion, the diet was the main stimulus ... As for the pain in my legs ... sometimes I have a bit of sciatica, but in the pool nothing hurts. We go to the pool twice a week, my wife and I. There has been a marked improvement in my health conditions. And the next thing is… to stop smoking” (ID02, male, age 61, W3).

## 4. Discussion

In an active ageing perspective, it has been recognized that to practice PA is beneficial both to older individuals (in terms of health status and well-being) and to society at large, so the promotion of PA in older age is a priority at the European policy level [2, 3, 91].

It is especially useful to study this issue in specific relation with the retirement transition, since the latter represents a turning point in each individual's life, for several reasons. For instance, for the need to reorganise the daily routine in light of the time usually spent at work, and or finding new functional needs to satisfy previously kinked at the professional life, such as identity and structure [26, 44].

Previous studies on the management of PA during the retirement transition evidenced mixed results, so it is still not clear how retirement shapes and affects PA behaviors of older individuals and there is no consensus on this issue [18]. Furthermore, few studies explored the gender issue in this respect (e.g., [59, 60]).

To start filling the gap in knowledge in this respect, the present three-year longitudinal study explored the management of PA during the transition from work to retirement of 24 women and 16 men, in Italy.

Answering to RQ1, this study demonstrated that the preferred PA both by women and men is walking along the transition to retirement. The latter is an activity which is relatively easy to carry out, with limited efforts to be spent in terms of preparation and equipment, so it is not surprising that it results as the main PA in this life phase. Moreover, data showed that, along the three study waves, several participants replaced harder and physically demanding activities with others, lighter [20] and that could be adapted to the different physical condition, such as less physical strength and resistance, and social needs (e.g., the need to replace the relationships they had at the work place with others), that they were experiencing during the transition to retirement. From this perspective, walking represented a chance for socialization, fun, and leisure [35], especially when it was practiced with a group of friends or with the partner. In light of the above, the analysis seems to suggest that having fun and enjoying PA can be a strong input and a further motivation for the acceptability of PA interventions to older adults [92], since they are in a life stage when fitness and aesthetic are not so important anymore, but healthy ageing represents a common objective. The aspect of fun connected to PA for older adults is still underestimated, and it would deserve more attention by scholars. Moreover, policy makers should consider, in line with Social Developmental Goal number 11 sustainable cities and communities [93], to increase the number of specific spaces devoted to walking in cities (where the larger part of older people live; [94]) in addition to bike paths. This would allow older people to increase PA in terms of walking, due to more opportunities offered.

The gym seems to be an activity preferred especially by women, in this phase of life, also seen as an opportunity for social networking (as it was found in an evaluation study in Cracow, Poland—[95]). The policy message in this respect would be to underline this aspect, while promoting PA through gym by women. Seasonal PA linked to water is also practiced by a considerable extent, by the respondents.

Through RQ2, given the qualitative longitudinal approach of the study, we explored the possible pathways in terms of PA experience, trying to go beyond the common exploration of increased or decreased PA explored through quantitative (and mostly cross-sectional) studies [96, 97].

A very useful theoretical approach, for investigating this issue, resulted to be the resource-based dynamic perspective on the retirement adjustment [53], which recognizes and accepts that the experience of PA levels during the retirement transition could not necessarily be in a way or another, but rather it could have different directions according to resources owned, and the change in resources implied by the retirement transition, in terms of, among others, social, emotional, physical, cognitive, and motivational resources. Moreover, the aim of this study was to gain a better understanding of the factors that lead older women and men to remain active in PA throughout the retirement transition. Building further on the insights of van Solinge and colleagues [31], planning activities when at work for after being retired gives a motivational insight. However, and more specifically, this study was designed to gain a better understanding of the extent to which adjustment and exploration mechanisms are important in explaining differences in PA both when one is still working in the labour market and when one has been retired for one year.

We found that the individuals' experience in terms of PA during the retirement transition can be of various types. These types may be led back to two main categories, one of them indicating that PA levels may benefit from the retirement transition (those individuals who incremented the level of PA or who at least maintained the same level), while the other indicating that PA levels may worsen in the transition to retirement, or be absent at all (i.e., the three groups of decreasers, fluctuaters, and inactive). This suggests that it may not be too useful to study this issue strictly in terms of activity, continuity, or disengagement theory [40, 43, 47], since all of these theories could fit with some of the types resulting from this study. However, to men the activity theory seems to adapt more than other theories, in which according to our results, they often exploit the more time availability characterizing the condition of retired individuals, to increase the level of PA. There is not a clear indication concerning this, with respect to women. However, still important, our results indicate that especially women may have the possibility to start practicing PA just once retired. This would mean that they, more than men, may encounter barriers to PA during the working life. Our results confirm that in the “familistic” Italy, informal care and work-life balance may still be an issue especially for women [98, 99], being potential barriers for their engagement in PA before retirement. In order to realise their life plans according to wishes and expectations, policies at the company and public policy levels should seriously deal with initiatives useful to help reconciling professional and informal care duties of working career, and more in general, with work-life balance issues, with particular attention to women.

Answering to RQ3, the results obtained in the present study in this respect allowed to identify the main drivers and the main barriers to PA during the retirement transition in terms of resources and the consequences of this in terms of behavioural profiles.

The more time availability resulting from the condition of retired individual is a driver especially for who generally practiced and cultivated PA in younger ages, already, and with no specific barriers, in terms of resources. PA in these cases was considered as a means for staying healthy during the previous life phases, and it remained a healthy habit. These individuals like practicing PA for reasons of health and well-being outputs and, especially women, to socialize and increase social networking (i.e., the categories of increasers and of starters). As also pointed out above, this motivational trigger should be highly considered, when promoting PA among older women.

It is confirmed that poor health conditions represent a main barrier to PA, during the retirement transition [57, 58, 76]. However, this study clarified that even in case of poor health, when PA is highly regarded in terms of social aspects linked to it, this barrier (depending of course by the level of physical impairment concerned) may not be fully penalizing, in terms of PA (i.e., continuers). Thus, promoting in a stronger way the social aspect of PA at the policy level, among older people with poor health, may be useful to increase the level of PA among the latter. Another motivational factor to continue practicing PA in spite of barriers is, especially for men, the PA effects on body shape. Thus, the result found by Beck and colleagues [59] in this respect could particularly apply to this group of older people.

The category of people with presumably the highest level of frustration relative to their PA experience, during the retirement transition, is that of people who continuously increases and/or decreases the level of PA (i.e., fluctuaters). The results of this study seem to indicate that they highly consider PA, they are motivated and would like to be among the “increasers” or at least among the “continuers;” however, they cannot fully realise what they wish, due to sudden and unexpected events that detract them from their PA aims: it could be a health problem, or it could be undesirable family duties in terms of elder caregiving or grandparenting. In the latter case, which apply especially to women, more efforts are needed at the policy level to defamilise the Italian currently still gendered welfare state [100, 101], in order to allow older women to have more freedom of choice regarding their life plans.

As for the categories of older people who reduced their level of PA or did not start at all this activity after retirement, the results of this study suggest that both for men and women, it could be mainly a matter of lack of (or decreasing) motivation (e.g., laziness) or interest. In these specific cases, more substantial efforts should be spent at the policy level, to sensitize these categories of older people about the benefits of PA, and to create opportunities to participate in initiatives of PA. Such opportunities might also be created through cost-effective digital platforms which may reach a considerable number of individuals [102]. For instance, virtual coaches and health technologies (e.g., wearable fitness devices and mobile health applications/devices) [61, 62] may play a role in stimulating and motivating older adults to identify and practice PA. In this respect, findings about the acceptability and effectiveness of web-based and virtual coaching interventions by older adults are encouraging [64, 103, 104], also to counteract the negative side-effects of pandemic-related policy measures, on older individuals' physical and mental well-being [105].

Overall, despite being important to study the specific life span of the retirement transition in this respect, given the importance of PA practice in previous life stages as driver of PA in older age, these findings call for appropriate and widespread awareness campaigns and policies highlighting the importance of PA for individual's health and well-being in a life-course perspective, from childhood and adulthood, to be put in place from schooling years and in the workplaces.

Especially workplaces deserve particular attention, in light of the barriers to PA encountered by women in these contexts, as above underlined. Also, training/measures are welcome, to sensitize people in the cusp of retirement about the importance of PA (even in later life) and motivate them to maintain/increase PA during retirement, for tackling some of the challenges associated with ageing (e.g., the loss of muscle mass, strength, and power, for which some specific methods/measures could be promoted, as for example those based on resistance training) [14, 15].

Overall, the fact that the effect of retirement on health and lifestyles of older adults, including PA behaviors, can be different for men and women, might also be due to different retirement rules, incentives, and statutory retirement ages applied to both genders in most countries [106]. However, since there is a scarcity of research and data concerning the possible role that different retirement age between women and men might play in affecting the PA engagement of older people, there is the need to promote further studies on this topic.

The main limitation of the present study is that the sample investigated is not representative of the whole population, so the findings obtained cannot be generalized. However, its qualitative longitudinal design allowed to analyse in-depth and in detail the management of PA by older people during the retirement transition, thus, resulting in a richness during data analyses. It would be interesting to study these findings would be confirmed by studies based on representative samples.

## 5. Conclusions

This paper adds to the literature especially by identifying specific behavioural types in relation to PA during the retirement transition and by associating to these type specific drivers and barriers to PA. This is in line with the call for more customized PA interventions for people experiencing the retirement transition [1, 107]. While policy making may benefit from these results, research suggests that top-down policy making in active ageing issues should be avoided, and that to apply a codecisional model involving all the relevant stakeholders may be useful [108].

A characteristic of this study is that it has been carried out in Italy, a country belonging to the Mediterranean welfare model [99]. In this country, the role of the family is crucial. Caring tasks are mainly carried out by family members, mostly women. This has a negative impact on female labour market participation [109]. Furthermore, no gradual retirement options exist in Italy, and this is in line with Italian older workers' aspirations, who basically do not want to work after retirement [5]. All this has an impact on the experience of PA, thus, future research should test whether the results of this study could be applied to countries belonging to different welfare regimes.

## Figures and Tables

**Table 1:** Baseline (before retirement) PA levels of the sample, by gender.

	No	Low	Medium	High	Total
Women	10	5	4	5	24
Men	3	10	2	1	16
Total	13	15	6	6	40

**Table 2:** Behavioural PA types of older adults in transition to retirement, by gender.

Behavioural types	Men	Women	Total
Increasers	9	6	15
Starters	0	5	5
Continuers	3	1	4
Fluctuaters	1	4	5
Decreasers	2	4	6
Inactive	1	4	5
Total	16	24	40

**Table 3:** Behavioural PA types of older adults in transition to retirement, by educational level.

Profile	Educational level			Total
	Low (ISCED 1-2)	Intermediate (ISCED 3-4)	High (ISCED 5+)	
Increasers	9	4	2	15
Starters	2	2	1	5
Continuers	2	1	1	4
Fluctuaters	0	5	0	5
Decreasers	2	4	0	6
Inactive	2	1	2	5
Total	17	17	6	40

**Table 4:** Main drivers of and barriers to PA for men and women after retirement.

Behavioural types	Main drivers of PA		Main barriers to PA	
	Men	Women	Men	Women
Increasers	PA is a healthy habit Physical well-being More time availability	PA is a healthy habit Socialization	/	/
Starters	N.A.	Willpower More time availability Companionship	N.A.	/
Continuers	Companionship	Companionship	Poor health	Poor health
Fluctuaters	Health benefits	Fun and leisure	Poor health	Caregiving
Decreasers	/	/	Laziness	Poor health
Inactive	/	/	Lack of interest in PA	Lack of interest in PA Poor health Caregiving

## Data Availability

Data used in this paper are available upon request to the authors.
